# Olanzapine Increases Neural Chemorepulsant—Draxin Expression in the Adult Rat Hippocampus

**DOI:** 10.3390/ph14040298

**Published:** 2021-03-27

**Authors:** Artur Pałasz, Aleksandra Suszka-Świtek, Jacek Francikowski, Marek Krzystanek, Katarzyna Bogus, Jakub Skałbania, John J. Worthington, Inga Mrzyk

**Affiliations:** 1 Department of Histology, Faculty of Medical Sciences in Katowice, Medical University of Silesia, 40-752 Katowice, Poland; aswitek@sum.edu.pl (A.S.-Ś.); kbogus@sum.edu.pl (K.B.); skalbaniakuba@gmail.com (J.S.); 2 Laboratory of Insect Physiology and Ethology, Institute of Biology, Biotechnology and Environmental Protection, Faculty of Natural Sciences, University of Silesia in Katowice, 40-007 Katowice, Poland; jacekfrancikowski@wp.pl; 3 Clinic of Psychiatric Rehabilitation, Department of Psychiatry and Psychotherapy, Faculty of Medical Sciences in Katowice, Medical University of Silesia, 40-635 Katowice, Poland; krzystanekmarek@gmail.com; 4 Division of Biomedical and Life Sciences, Faculty of Health and Medicine, Lancaster University, Lancaster LA1 4YQ, UK; j.j.worthington@lancaster.ac.uk; 5 Łukasiewicz Research Network—Institute of Industrial Organic Chemistry, Branch Pszczyna, 43-200 Pszczyna, Poland; imrzyk@ipo-pszczyna.pl

**Keywords:** draxin, olanzapine, hippocampus, adult neurogenesis

## Abstract

Draxin belongs to the family of inhibitory axon-guiding factors that regulate neuronal migration and axonal spreading in the developing brain. This glycoprotein has recently been considered to play an important role both in hippocampal differentiation and adult neurogenesis in the dentate gyrus. Given that it has been reported that antipsychotic drugs may affect neurite growth and neurogenesis, we have therefore investigated whether chronic treatment with olanzapine modulates draxin immunoreactivity in the adult rat hippocampus. After analysis of local fluorescence intensity, we found a significant increase of draxin immunoexpression both in the subgranular zone (SGZ) and granular zone of the rat hippocampus following long-term olanzapine administration. This study reveals, for the first time, the modulatory effect of the atypical antipsychotic medication olanzapine on expression of the novel chemorepulsive protein draxin in the context of adult neurogenesis regulation. Moreover, this is the first report dealing with pharmacological aspects of draxin signaling. An elevated draxin expression may indirectly support a recently formulated hypothesis that olanzapine may drive adult neurogenesis via paracrine draxin-related signaling. This action of draxin is a new element in the neurogenesis mechanism that may be part of the action of second-generation antipsychotics in the treatment of schizophrenia, indicating more detailed molecular studies are urgently required to fully investigate these potential novel mechanisms of neurogenesis.

## 1. Introduction

Draxin (dorsal repulsive axon guidance protein, neucrin) is an inhibitory axon guiding factor and local chemorepulsive glycoprotein involved in neuronal migration and neurite growth in the developing brain [1,2,3]. This relatively novel regulatory protein is considered to play an important role in hippocampal differentiation, as the anterior part of this cortical structure is highly underdeveloped in draxin knockout mice due to enhanced granule cell apoptosis [4]. Draxin seems to also be necessary for normal development of the interhemispheric connections and spinal-cord organization [5], with lack of this protein causing absence of brain commissural pathways, as well as defasciculation of spinal-cord fibers [6]. Draxin expression is also found postnatally in several brain structures such as the hippocampus, olfactory bulb, cortex, midbrain, cerebellum, and pontine nuclei. Human draxin comprises 349 amino acids (58 kDa), and contains a signal peptide and 10 cysteine residues in the conserved C-terminal region [7]. An important role of draxin in hippocampal adult neurogenesis has been recently identified [4,8]. Late Tbr2 and NeuroD1-expressing neural progenitors are considered to release draxin in a paracine manner [9]. This regulatory protein is involved in canonical Wnt/β-catenin signaling as a ligand of LRP5/6 (low-density lipoprotein receptor-related protein) and Frizzled receptors [10,11], and can also attenuate apoptosis of neuroblasts in the subgranular zone (SGZ) via netrin DCC (deleted in colorectal cancer) receptor. Indeed, draxin knockout mice show disturbed proliferation and differentiation of neural stem/progenitor cells in the dentate gyrus [9]. Neurogenesis, neuroprotection, and cell death are related to the mechanism of action of second-generation antipsychotic drugs (SGAs) [12,13]. Research confirms the possibility of SGA-stimulated neurogenesis, mediated via multiple molecular mechanisms [12]. The mechanisms involved in neurogenesis caused by SGAs are not fully understood, and getting to know them may help in developing more effective strategies for schizophrenia treatment. Olanzapine is one of the most effective and most frequently used second-generation antipsychotic drugs in the treatment of schizophrenia [14]. Olanzapine in schizophrenia is effective mainly in relation to positive symptoms, but it also has a beneficial effect on negative symptoms [15]. Olanzapine, like other second-generation antipsychotics, is less effective for cognitive dysfunctions, which may be associated with changes in the composition of NMDA receptors [16] caused by them. Despite its high clinical effectiveness, its use is limited by the occurrence of metabolic disorders (weight gain, dyslipidemia, and diabetes mellitus) [14]. Among other side effects, somnolence, prolactin elevation, anticholinergic side effects, and QTc prolongation greater than placebo are the most common [14].

Olanzapine, an atypical antipsychotic agent, acts as an antagonist of brain dopaminergic receptors (D1-D5), with some affinity to others, including serotoninergic (5-HT2A/2C, 5-HT3, 5-HT6), α1-adrenergic, muscarinic, and histaminergic [17]. Olanzapine reduces both negative and positive schizophrenia symptoms via selective silencing of mesolimbic dopaminergic neurons, without depression of striatal neuronal circuits involved in motor functions [18,19]. In the studies conducted on rats, olanzapine stimulates cellular proliferation in the subventricular zone (SVZ) and brain-derived neurotrophic factor (BDNF) expression in the hippocampus [20,21]. The stimulatory effect on adult neurogenesis and neuroprotection may be responsible for the beneficial effects obtained clinically in patients treated with olanzapine. It has been shown that olanzapine preserves the brain volume in first episode of schizophrenia [22]. These results were confirmed in the meta-analysis by Vita et al. [23]. It has shown that, unlike people treated with first-generation antipsychotics, patients treated with SGAs, including olanzapine, do not have significant gray-matter loss, and there is even a trend toward increasing the volume of gray matter in the frontal and temporal lobes [23]. Regarding recent studies reporting that antipsychotic drugs may affect axonal development and neurogenesis [24,25,26], we have investigated whether long-term treatment with olanzapine modulates draxin immunoreactivity in the adult rat hippocampus. In the present article, we show for the first time an effect of antipsychotic drug administration via olanzapine on draxin expression in the canonical SGZ zone of the rat dentate gyrus.

## 2. Results

**Figure 1:**
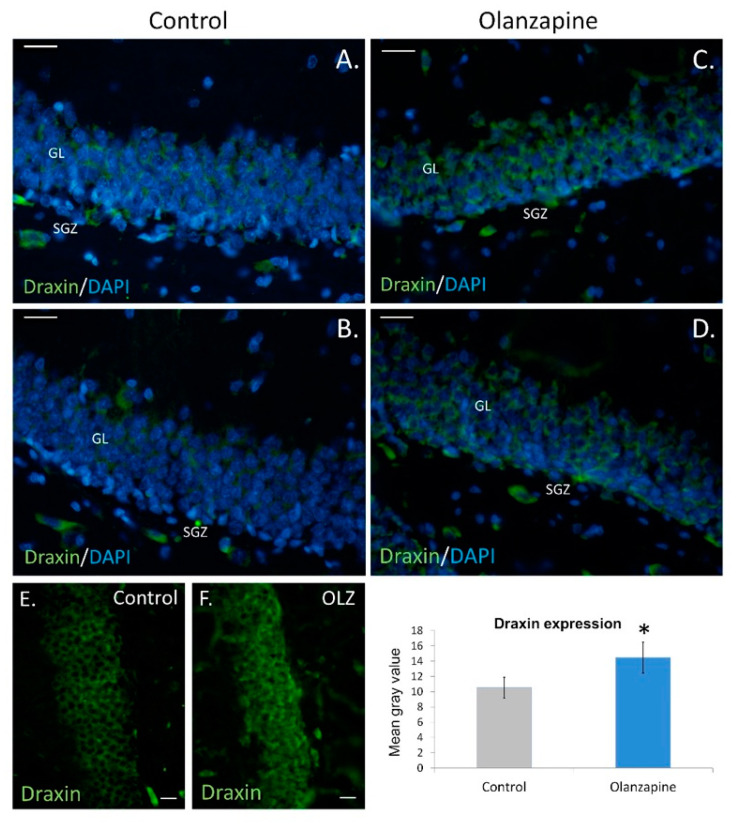
Draxin expression in the rat hippocampus. Control (**A**,**B**), olanzapine (**C**,**D**). Microphotographs show granular layers (GL) and subgranular zones (SGZ) of dentate gyrus with disperse draxin fluorescence. Scale bars: 20 μm (**A**–**D**), 10 μm (**E**,**F**). Mean gray value of sections examined in the study + standard error of the mean (SEM), * *p* < 0.05.

Draxin gene deletion causes severe dysregulation of this process within the SGZ in rats [9]. Here, we have demonstrated a significant increase of draxin immunoexpression both in the SGZ and granular zone of the rat hippocampus after long-term treatment with olanzapine. Mean staining/fluorescence intensity was higher in the neuroleptic-treated group than in the control group (*p* = 0.0005; F, DFn, Dfd: 2.322, 6, 6). In the analyzed brain sections, draxin immunoexpression was very dispersed within the subgranular and granular layers of the dentate gyrus, with diverse intensity of the fluorescent signal (Figure 1). This highly diffused reaction may speak for the paracrine manner of draxin release by special cell populations in the dentate gyrus.

## 3. Discussion

It was recently suggested that draxin may be necessary for the origin and/or differentiation of neural stem cells and progenitors during hippocampal adult neurogenesis [9]. Previous reports revealed that chronic olanzapine administration supports the origin of Sox-2 and DCX in BrdU-expressing cells in the rat SGZ and SVZ sites [27,28], and also DCX in Ki67-positive cells in the rat hypothalamic noncanonical subependymal region [29]. Our results suggest indirectly and cautiously that olanzapine may promote adult neurogenesis via stimulation of draxin expression and its signaling in the rat hippocampus. However, no significant changes in the number of hippocampal TUC-4-expressing cells were found. On the other hand, these data are also in line with a study reporting a stimulatory and neuroprotective effect of olanzapine on neuregulin-1 (NRG-1) expression in rats with PCP-induced failure of axonal spreading and synaptogenesis [30]. Upregulation of NRG-1-signaling may be therefore involved in the potential olanzapine-related improvement of some cognitive deficits. The hyperactivity of the dopamine D_2_ receptor in the rat pyramidal cortex causes axonal spreading impairment via decrease of pGSK3β signaling by the complex D_2_R-DISC1 [25]. Interestingly, the classical and atypical neuroleptics aripiprazole and haloperidol may reduce this effect and prevent neurite lesions [25]. On the other hand, in mice prenatally exposed to neuroleptics, haloperidol and risperidone decreased proliferation and differentiation of neural progenitors in SGZ and blocked dendrite branching of granular cells. However, in the case of haloperidol, but not risperidone, an impaired dendritic elongation and reduced spine number of local neurons occurred [24]. Thus, haloperidol, a classical antipsychotic drug, seems to be more harmful to prenatal hippocampal development than the atypical one—risperidone. The selective serotonin reuptake inhibitor fluvoxamine may also restore NGF-induced axonal growth after its blockade caused by dexamethasone in vitro; the effects probably are caused by phosphorylation of p-Akt and stimulation of σ1 receptor [31].

**Figure 2:**
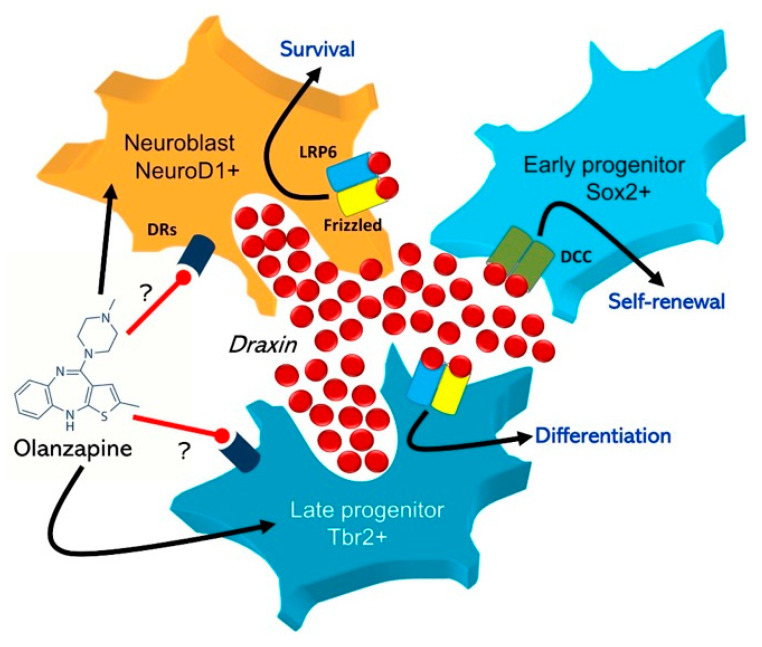
Hypothetical mechanism of possible olanzapine effect on draxin-releasing cells in the hippocampal stem cell niche. Expression and TUC4/NeuroD1-positive neuroblasts are considered as the main source of draxin in the dentate gyrus. Draxin secreted into the intercellular environment binds to membrane LRP6/Frizzled receptors of both aforementioned cells, and to DCC receptors of Sox2/nestin expressing early progenitors that support their proliferation, differentiation, and self-renewal within the SGZ niche.

The mechanism of olanzapine action on draxin-releasing cells including Tbr2/NeuroD1-expressing late neural progenitors in the SGZ is not clear. Studies reporting effects of dopaminergic agonists/antagonists on hippocampal adult neurogenesis suggest very indirectly that these cell populations may express dopamine receptors (Figure 2). For instance, a blockade of D_2_ receptors with haloperidol promoted adult neurogenesis by enhancement of neural-progenitor generation in the rat SGZ [32]; however, no effect of dopamine modulators on this process was reported [33]. Conversely, another study showed stimulatory effect of D_2_ agonist on adult neurogenesis in mouse SGZ/SVZ in vitro [34]. The complete receptor profile of neural stem and progenitor cells remains understudied, which makes it currently impossible to formulate any convincing conclusions. Possibly, the enhancement of draxin expression in the adult hippocampus after long-term treatment with olanzapine may reflect the same neuroleptic effect during developmental neurogenesis. Atypical antipsychotic administration in early prenatal life may potentially disturb cerebral cortex histogenesis and neural circuit organization [35]; however, a potential role of chemorepulsive draxin signaling in this effect remains unknown. A confirmation of draxin neurochemistry in the context of pharmacomodulation requires further studies; e.g., quantification of the peptide concentration with Western blotting or mRNA-level assessment. Despite the limitations of our study, it may cautiously suggest that draxin can be considered as novel and potentially important regulatory molecule of the brain.

## 4. Materials and Methods

Studies were carried out on adult (2–3 months old, 180–220 g) male Sprague–Dawley rats from the Medical University of Silesia Experimental Center housed at 22 °C with a regular 12/12 light–darkness cycle with access to standard Murigran chow and water ad libitum. All procedures were approved by the Local Bioethic Committee at the Medical University of Silesia (decision no. 36/2012) and were conducted in a manner consistent with NIH Guidelines for Care and Use of Laboratory Animals.

Two groups of rats (5 individuals each) received control vehicle or olanzapine (5 mg/kg/day, dissolved in isotonic saline) by intraperitoneal injection for 4 weeks. This optimal, nontoxic dose was established on the basis of pharmacological standards developed in preclinical studies on antipsychotics in the context of adult neurogenesis [27,29]. 24 h after the last drug administration, rats were quickly anaesthetized with isoflurane and then immediately sacrificed by decapitation. Rat brains were excised, fixed with 4% paraformaldehyde PBS (pH 7.2–7.4), dehydrated, embedded in paraffin, and finally sectioned on the microtome (Leica Microsystems, Mannheim, Germany) in the coronal planes for SGZ (−2.00 to −2.80 mm from bregma) at 7 μm-thick slices. The distance between 10 sections used per animal was 50 μm. After blocking with 5% goat serum, sections were incubated overnight with the rabbit antirat draxin polyclonal antibody (1:1000, Biorbyt Ltd., Saint Louis, MO, USA; orb 314002). After incubation with the aforementioned primary antibodies, all brain sections were kept in darkness with secondary antibodies labeled with FITC (1:200, Abcam) and then mounted on slides with DAPI-containing medium.

For morphometric assay of draxin immunoreactivity, 5 sections per rat were used. Due to the highly dispersed and diffuse draxin immunofluorescence in densely arranged granular cell clusters, the number of cells was not calculated. All images (2 per section) were captured with Nikon Coolpix optic systems and processed using Image ProPlus software (Media Cybernetics, Rockville, MD, USA). Histologically analogous serial sections were analyzed densitometrically with use of ImageJ (v1.51j8). Pictures were normalized and background was extracted (rolling ball radius: 100 pix). Further, the green channel was isolated as a BW (8-bit) picture. At this stage, five ROIs (250 × 250 pix) were selected in the region of staining to calculate mean grey value, then the average staining intensity was calculated for the picture. For control and treated groups, seven pictures were analyzed (mean values were obtained from this assay). The average gray value within the selection was the sum of the gray values of all the pixels in the selection divided by the number of pixels, reported in calibrated units (e.g., optical density) if Analyze&gt; Calibrate was used to calibrate the image. For RGB images, the mean was calculated by converting each pixel to grayscale using the formula gray = 0.299 red + 0.587 green + 0.114 or: gray = (red + green + blue)/3 if " Unweighted RGB to Grayscale Conversion" is checked in Edit&gt; Options&gt; Conversions. The total number of TUC-4-positive cells in the comparable SGZ areas was additionally estimated. Cells were counted from standardized frames of the specimen (5 section per rat, 2 frames per section). Statistical analyses were performed using Statistica (Systat Software, San Jose, CA, USA). Mean differences between the groups were analyzed using an unpaired two-tailed t-test and a nonparametric Kruskall–Wallis test. Differences were considered statistically significant at *p* ≤ 0.05.

## 5. Conclusions

We have shown for the first time an increase of draxin immunoexpression in the adult rat dentate gyrus after long-term treatment with the atypical antipsychotic medication olanzapine. This may indirectly support a recently formulated suggestion that olanzapine may increase adult neurogenesis in both the canonical and hypothalamic sites via paracrine draxin-related signaling; however, further molecular studies are urgently required to confirm this possible regulatory interplay.

## Data Availability

The data reported in this study are available in this manuscript.
